# Supplementary material for: Identification of the DPP-IV Inhibitory Peptides from Donkey Blood and Regulatory Effect on the Gut Microbiota of Type 2 Diabetic Mice
Source: Foods. 2022 Jul 20;11(14):2148. doi: 10.3390/foods11142148 (PMC9316604; doi:10.3390/foods11142148)
Supplement: Supplementary file 1 [file foods-11-02148-s001.zip › foods-1791938-supplementary.pdf]

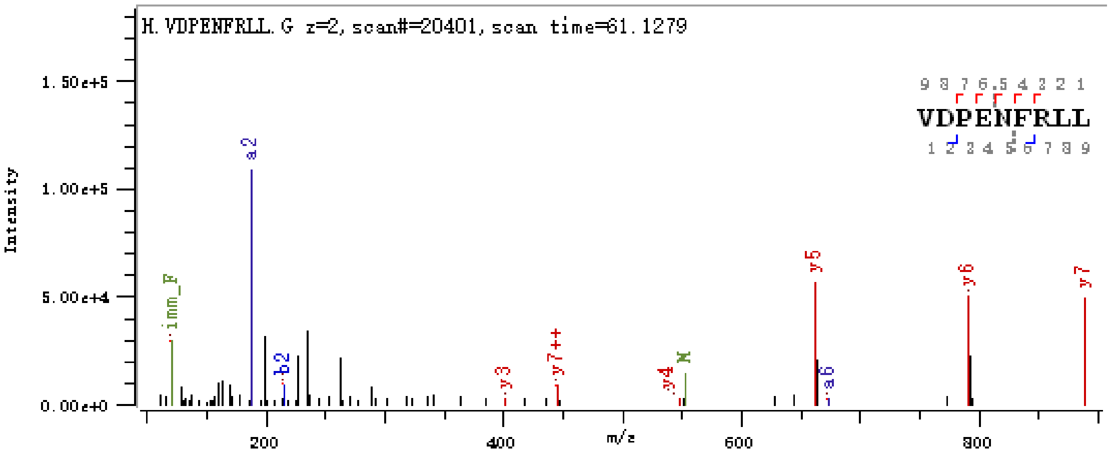

Figure S1. secondary MS of active peptide VDPENFRLL

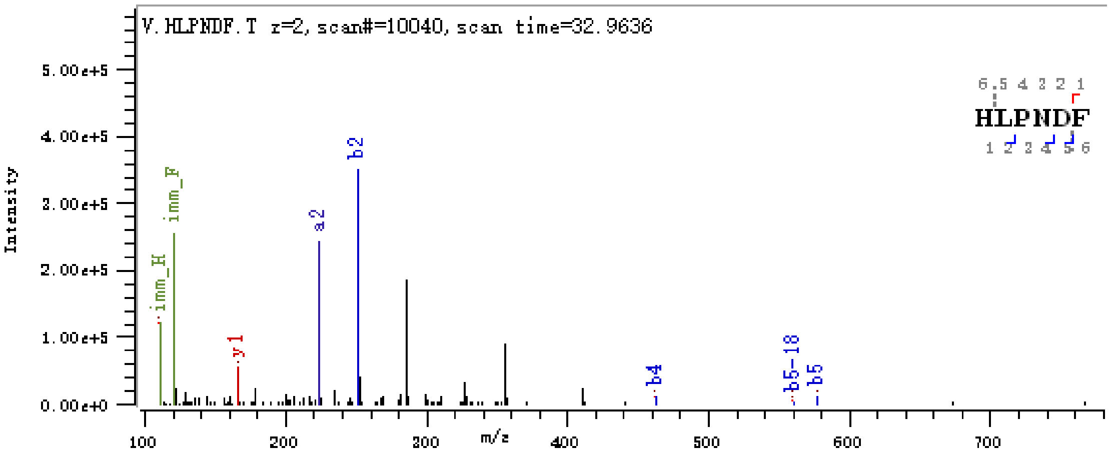

Figure S2 secondary MS of active peptide HLPND

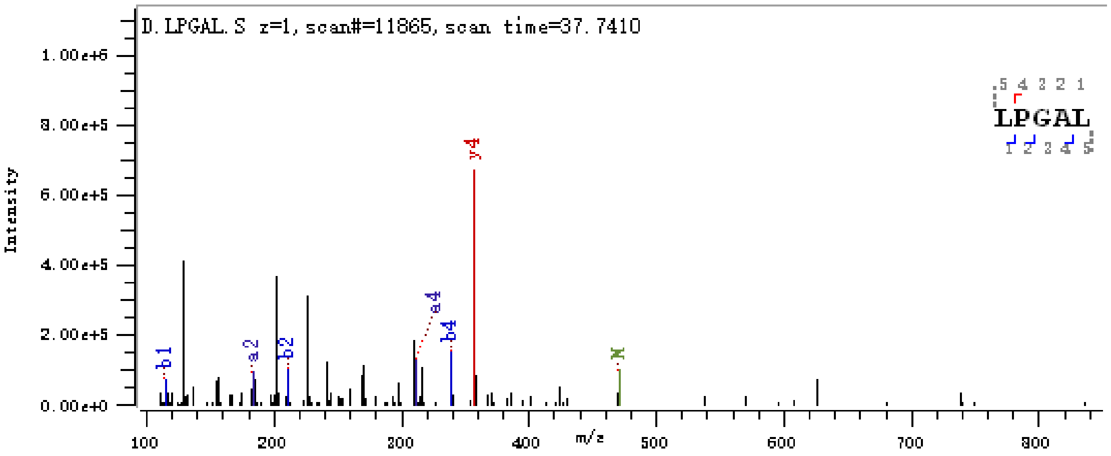

Figure S3 secondary MS of active peptide LPGAL

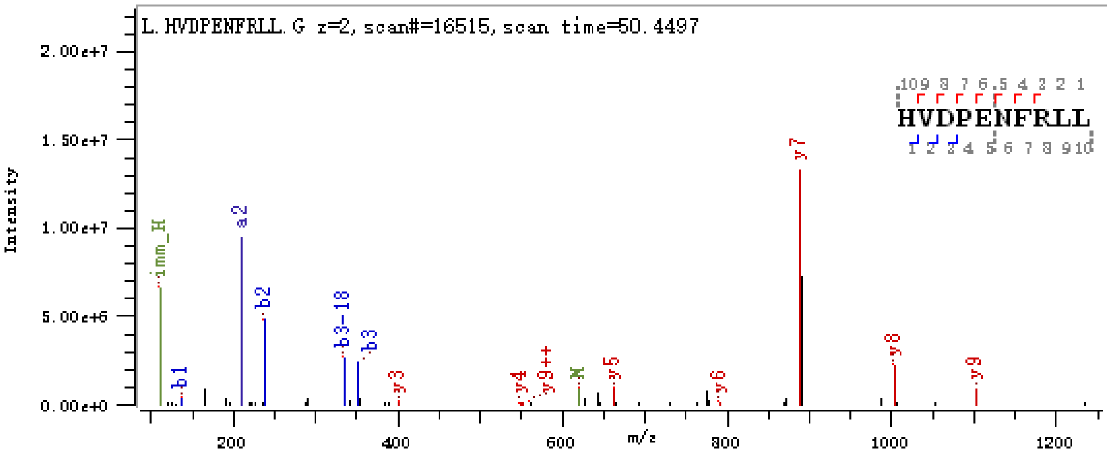

Figure S4 secondary MS of active peptide HVDPENFRLL

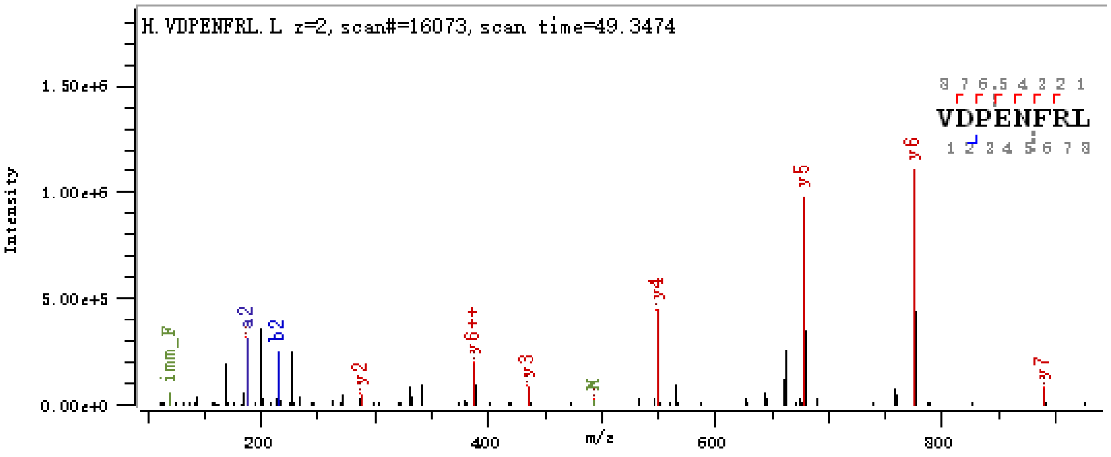

Figure S5 secondary MS of active peptide VDPENFRL

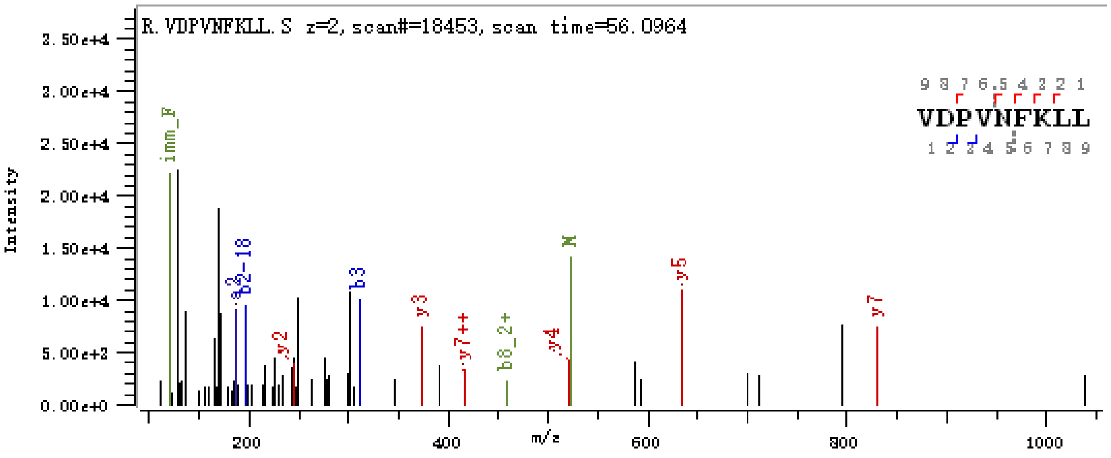

Figure S6 secondary MS of active peptide VDPVNFKLL

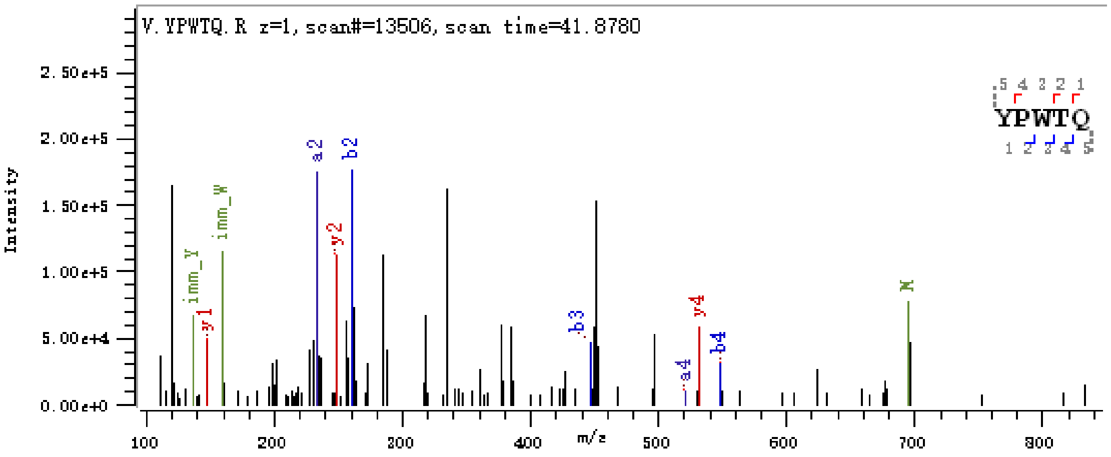

Figure S7 secondary MS of active peptide VDPVNFKLL

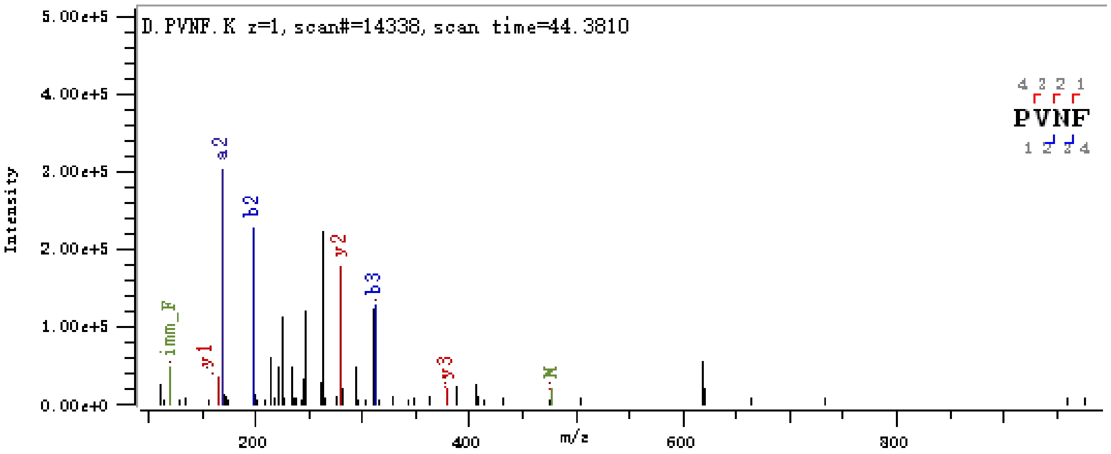

Figure S8 secondary MS of active peptide PVNF

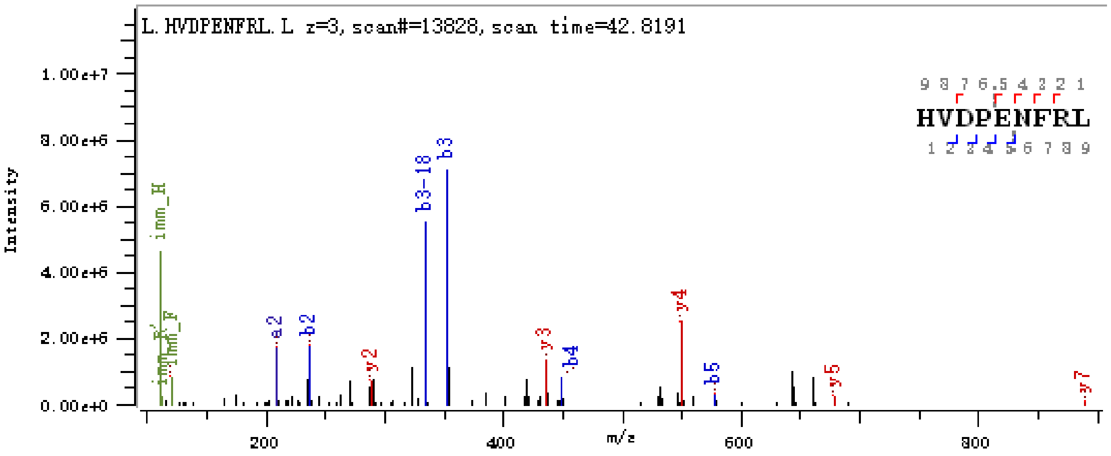

Figure S9 secondary MS of active peptide HVDPENFRL

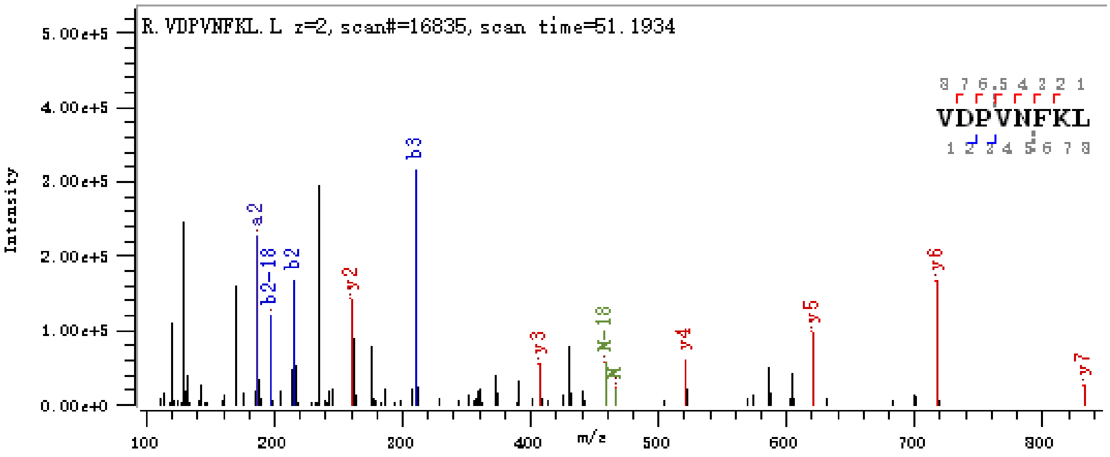

Figure S10 secondary MS of active peptide VDPVNFKL

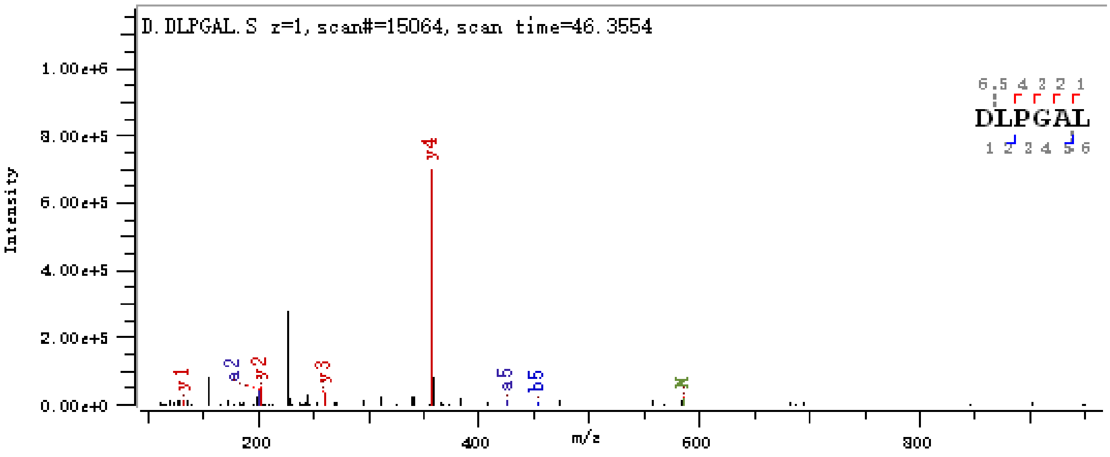

Figure S11 secondary MS of active peptide DLPGAL

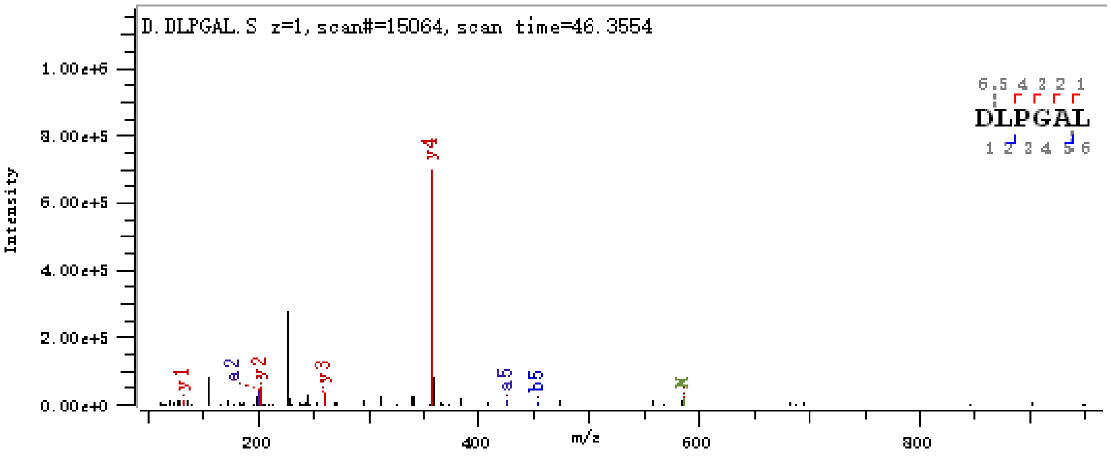

Figure S12 secondary MS of active peptide PHFDLS

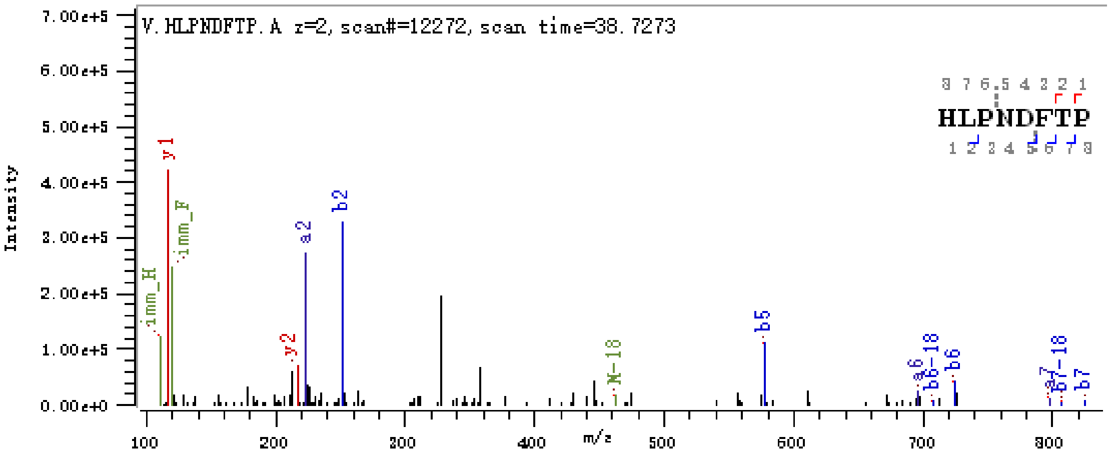

Figure S13 secondary MS of active peptide HLPNDFTP

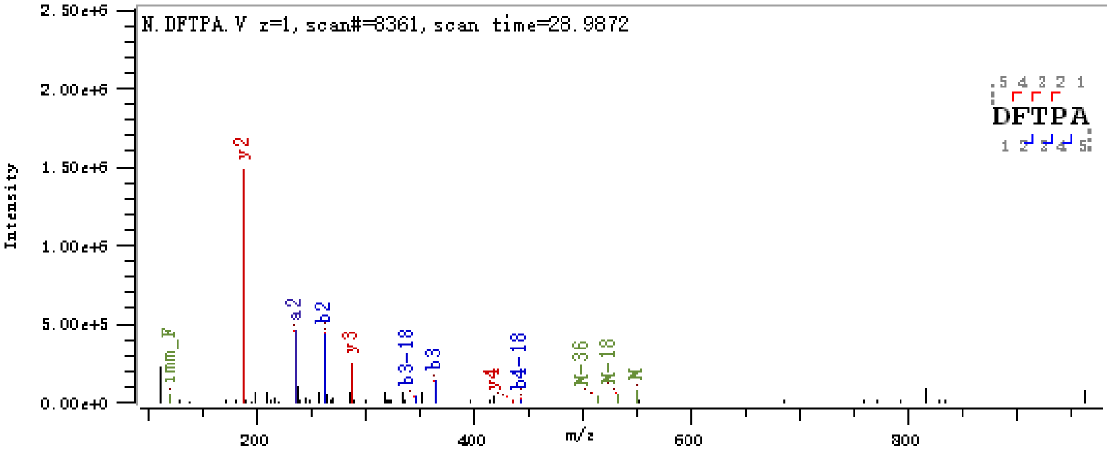

Figure S14 secondary MS of active peptide DFTPA

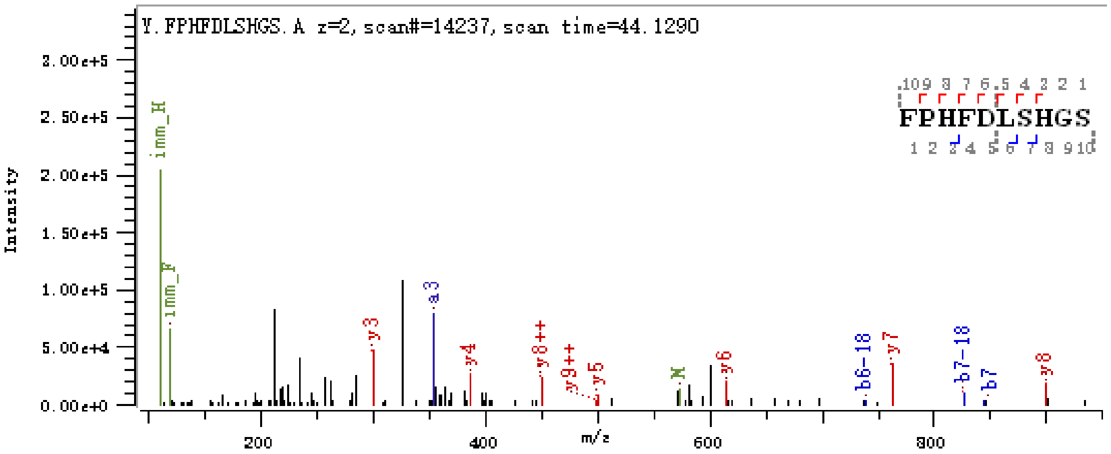

Figure S15 secondary MS of active peptide FPHFDLSHGS

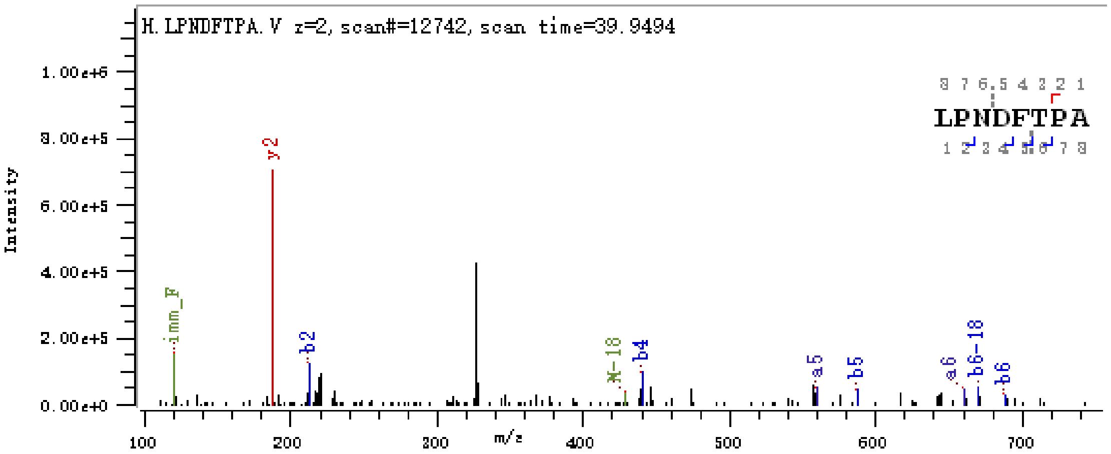

Figure S16 secondary MS of active peptide LPNDFTPA
